# Intra-individual physiological response of recreational runners to different training mesocycles: a randomized cross-over study

**DOI:** 10.1007/s00421-020-04477-4

**Published:** 2020-09-12

**Authors:** Peter Düking, Hans-Christer Holmberg, Philipp Kunz, Robert Leppich, Billy Sperlich

**Affiliations:** 1 Integrative and Experimental Exercise Science and Training, Department of Sport Science, University of Würzburg, Würzburg, Germany; 2 Swedish Winter Sports Research Centre, Department of Health Sciences, Mid Sweden University, Östersund, Sweden; 3 Department of Physiology and Pharmacology, Biomedicum C5, Karolinska Institutet, Stockholm, Sweden; 4 Chair of Software Engineering, Department of Computer Science, University of Würzburg, Würzburg, Germany

**Keywords:** Cardiorespiratory fitness, Endurance, Personalized training

## Abstract

**Purpose:**

Pronounced differences in individual physiological adaptation may occur following various training mesocycles in runners. Here we aimed to assess the individual changes in performance and physiological adaptation of recreational runners performing mesocycles with different intensity, duration and frequency.

**Methods:**

Employing a randomized cross-over design, the intra-individual physiological responses [i.e., peak (${\dot{\text V}}{\text O}_{2 {\rm peak}}$) and submaximal (${\dot{\text V}}{\text O}_{2 {\rm submax}}$) oxygen uptake, velocity at lactate threshold**s** (V_2_, V_4_)] and performance (time-to-exhaustion (TTE)) of 13 recreational runners who performed three 3-week sessions of high-intensity interval training (HIIT), high-volume low-intensity training (HVLIT) or more but shorter sessions of HVLIT (high-frequency training; HFT) were assessed.

**Results:**

${\dot{\text V}}{\text O}_{2 {\rm submax}}$, V_2,_ V_4_ and TTE were not altered by HIIT, HVLIT or HFT (*p* > 0.05). ${\dot{\text V}}{\text O}_{2 {\rm peak}}$ improved to the same extent following HVLIT (*p* = 0.045) and HFT (*p* = 0.02). The number of moderately negative responders was higher following HIIT (15.4%); and HFT (15.4%) than HVLIT (7.6%). The number of very positive responders was higher following HVLIT (38.5%) than HFT (23%) or HIIT (7.7%). 46% of the runners responded positively to two mesocycles, while 23% did not respond to any.

**Conclusion:**

On a group level, none of the interventions altered ${\dot{\text V}}{\text O}_{2 {\rm submax}}$, V_2_, V_4_ or TTE, while HVLIT and HFT improved ${\dot{\text V}}{\text O}_{2 {\rm peak}}$. The mean adaptation index indicated similar numbers of positive, negative and non-responders to HIIT, HVLIT and HFT, but more very positive responders to HVLIT than HFT or HIIT. 46% responded positively to two mesocycles, while 23% did not respond to any. These findings indicate that the magnitude of responses to HIIT, HVLIT and HFT is highly individual and no pattern was apparent.

## Introduction

The three physiological characteristics thought to be the primary determinants of running performance (Midgley et al. 2007) are commonly tested in the laboratory: (i) the maximal capacity to take up, transport and utilize oxygen (i.e., peak oxygen uptake [${\dot{\text V}}{\text O}_{2 {\rm peak}}$]); (ii) the ability to maintain high speed without accumulating lactate (Midgley et al. 2007) (the lactate threshold); and (iii) running economy (often expressed as the oxygen utilized while running at a given constant speed). To improve these variables, runners either enhance their volume of exercise per session (high-volume low-intensity training (HVLIT) at a blood lactate concentration < 2 mmol·l^−1^), increase the intensity of the exercise (e.g., in the form of high-intensity interval training (HIIT)) and/or train more sessions per week (HFT).

There are pronounced inter-individual differences in the physiological adaptations to different forms of training, with as many as 20% exhibiting no adaptation at all (Timmons et al. 2010; Montero and Lundby 2017; Garber et al. 2011). The explanation for such variability may include, among other things, genetic factors, different extents of compliance to a training schedule, and/or inappropriate exercise for the individual in question and/or his/her training status (Tanaka 2018; Joyner and Lundby 2018; Garber et al. 2011). While genes are thought to play a key role in determining, e.g., ${\dot{\text V}}{\text O}_{2 {\rm peak}}$ (Bouchard et al. 1999), emerging evidence indicates that the appropriate individual training dose can reduce the numbers of low- and non-responders to exercise (Montero and Lundby 2017). Zinner and colleagues (2018) found that the number of non-responders among recreational runners performing a 3-week mesocycle of training is lower when this training is in the form of HVLIT rather than HIIT or a combination of both. Unfortunately, this study compared groups and intra-individual responses to the different forms of training were not evaluated. Montero and Lundby (2017) showed that individuals who did not respond to 6 weeks of 1–5 60-min sessions of cycling per week did respond with two additional sessions per week (Montero and Lundby 2017). These investigators concluded that with a sufficiently large training load, all subjects improve their cardiorespiratory fitness (including, e.g., peak oxygen uptake and maximal power output) (Montero and Lundby 2017). However, a training load as extensive as theirs might not be feasible or even safe for all (amateur) athletes (Schwellnus et al. 2016; Soligard et al. 2016).

To our knowledge, no randomized cross-over design has yet been employed to assess intra-individual physiological responses to different forms of training with a comparable load.

We hypothesize that different forms of mesocycles in runners with a comparable load (i) induce different performance and physiological adaptations on group level and (ii) that each individual shows distinct adaptation to different mesocycles.

Accordingly, the aim of the present investigation was to assess the intra-individual responses of recreational runners (peak oxygen uptake, velocity at lactate threshold**s**, running economy, and performance (time-to-exhaustion)) performing HIIT, HVLIT or HFT.

## Methods

### Participants

Of the 32 participants who began the study, none of whom were competitive athletes, 13 (5 men and 8 women, initial ${\dot{\text V}}{\text O}_{2 {\rm peak}}$: 43.9 ± 5.2 ml·kg^−1^·min^−1^, age: 29 ± 3 years, height: 169 ± 6 cm, weight: 70.3 ± 9.5 kg (mean ± SD) completed all mesocycles of training and all testing procedures. The others dropped out because of time constraints. All participants provided their written informed consent to participate in this study, which was pre-approved by the ethical committee of the Department of Sport Science, University of Würzburg and performed in accordance with the Declaration of Helsinki.

## Experimental design

**Fig. 1:**
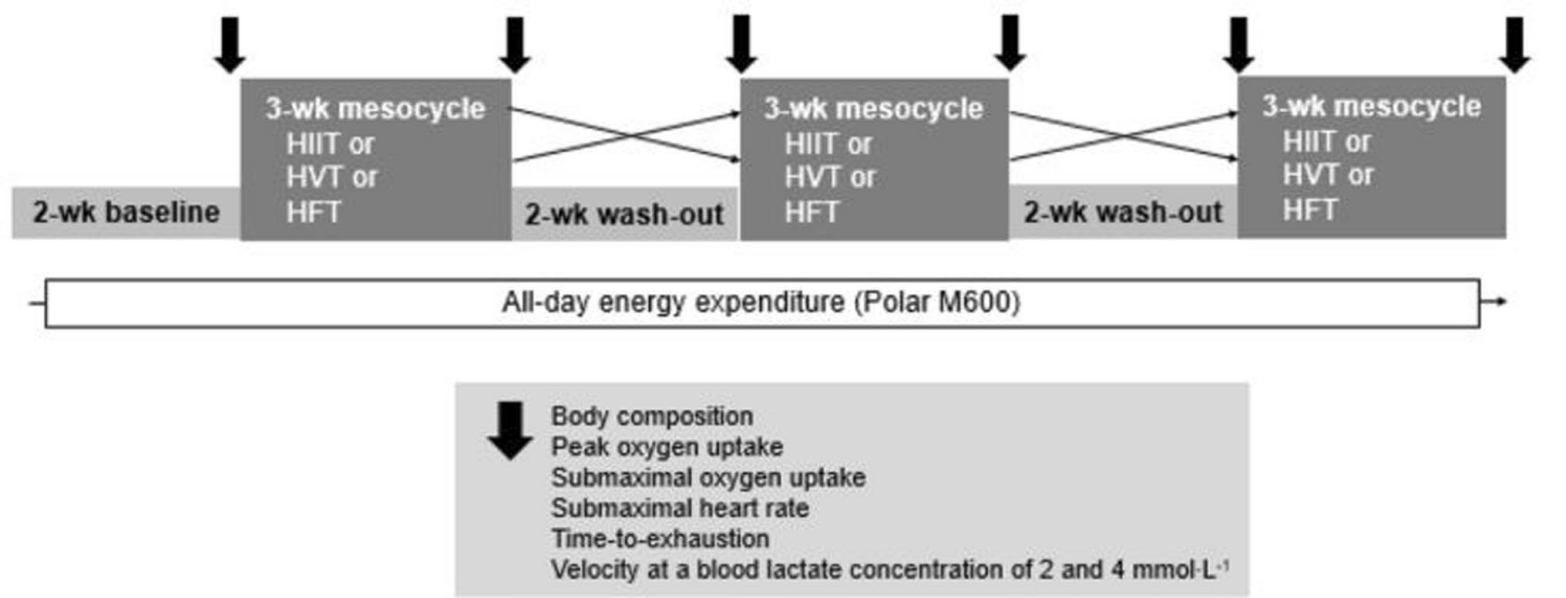
The overall study design including different mesocycles of training and parameters assessed during the 15 week cross-over experiment

Figure 1 illustrates the overall randomized cross-over experimental set-up and all variables assessed during training and laboratory testing.

The 15-week experiment involved six visits to the laboratory. Each participant first completed a 2-week baseline period, during which they monitored their routine training load using TRIMP calculations, as explained in detail below. Following this baseline period, each performed three different three-week mesocycles of training (HIIT, HVLIT and HFT) in randomized order and separated by a 2-week “wash-out” period with little or no training. The intensity of each training session was regulated relative to the peak heart rate determined in connection with the first laboratory visit.

A 3-week training mesocycle was chosen based on the findings by Zinner and colleagues (2018) demonstrating that 3-weeks of training suffice to elevate performance and physiological adaptations in recreational runners. A 2-week wash-out period with no or little training was chosen (i) to ensure compliance and adherence by narrowing down the overall study duration and (ii) since (depending on the population and specific training) 2–4 weeks of training interruption reverses previously gained training adaptation (Sousa et al. 2019).

Before and after each mesocycle, all performed an incremental all-out test in the laboratory designed to assess ${\dot{\text V}}{\text O}_{2 {\rm peak}}$, time-to-exhaustion (TTE), submaximal oxygen uptake (${\dot{\text V}}{\text O}_{2 {\rm submax}}$) and heart rate (HR_submax_), and running velocity at blood lactate concentrations of 2 (V_2_) and 4 (V_4_) mmol·l^−1^.

## Testing procedures

All participants were requested to refrain from consuming alcohol or caffeine for at least 24 h prior to all testing and to arrive at the laboratory well hydrated. Testing took place on approximately the same time of day to avoid disturbances of circadian rhythmic.

All testing included an assessment of fat-free mass, as well as an incremental running test to exhaustion (5 min at 1.94 m·s^−1^ (women) or 2.22 m·s^−1^ (men) on an incline of 1% with an incremental increase in speed of 0.36 m·s^−1^ once every 3 min and 15 s of passive recovery prior to each such increase for blood sampling) on a motorized treadmill (Mercury, h/p/cosmos sports and Medical GmbH, Nussdorf-Traunstein, Germany). To ensure maximal effort, verbal encouragement was given during incremental testing.

Both heart rate and oxygen uptake were monitored continuously using an open breath-by-breath gas analyzer (Cortex Metamax 3B, Leipzig, Germany), which was calibrated prior to each test with high-precision gas and a 3-L syringe, giving a maximal error of 2% (Macfarlane and Wong 2012). All gas analyses were divided into successive 30-s windows. Oxygen uptake was considered to have peaked when three of the following four criteria were met: (1) a respiratory exchange ratio > 1.1; (2) a plateau in ${\dot{\text V}}{\text O}_{2}$ (i.e., an elevation of ≤ 1.0 ml·min^−1^·kg^−1^ as the velocity was increased); (3) a heart rate within 5% of the age-predicted peak (HR_peak_); (4) and a peak capillary blood lactate concentration > 6 mmol·l^−1^. The highest 5-s average heart rate was considered to be HR_peak_. The lactate threshold was designated as 2 mmol·l^−1^. ${\dot{\text V}}{\text O}_{2 {\rm submax}}$, a surrogate for running economy, was defined as the average oxygen uptake during the last 60 s of a 5-min run at 1.94 (women) or 2.22 (men) m·s^−1^.

Capillary blood was sampled from the right earlobe during the 15-s recovery period prior to each incremental increase in speed, as well as after exhaustion was reached, for analysis of lactate with a handheld device (Lactate Pro 2, Arkray KDK, Kyoto, Japan). Running velocity at a capillary lactate concentration of exactly 2 mmol·l^−1^ (V_2_) was calculated by extrapolation between the running velocities immediately prior to and after this concentration was reached.

### 24-h monitoring of energy expenditure

Since concomitant extensive daily physical activity may improve responses to training (Hautala et al. 2012), we also monitored physical activity when the participants were not training. For this purpose each individual wore a multisensory device (Polar M600, Kempele, Finland) on the wrist for the entire experimental period, i.e., both during all training and wash-in phases, removing this device only for charging. The Polar M600, which has been validated with moderate correlation to a ActiGraph GT3X under free-living conditions (Degroote et al. 2017), recorded the intensity of activity continuously. To conform to established classifications (Ainsworth et al. 2011; Sedentary Behaviour Research 2012), we divided up energy expenditure (in min·d^−1^) as follows: sedentary (< 1.5 metabolic equivalent of task [MET])), or light (1.5–3 MET), or moderate (3–6 MET) or vigorous (> 6 MET).

### Training load

The participants’ heart rate (HR) during training was recorded by the multisensory device that was used for the 24-h monitoring of energy expenditure. Data was stored online (Polar Flow Software, Polar Electro OY, Kempele, Finland). As previously (Zinner et al. 2018), HR zones were defined as those associated with blood concentrations below, between, or above 2 and 4 mmol·l^−1^. The time spent in each zone during each training session was calculated. In addition, all participants rated their perceived exertion during each training session on the 6–20 Borg scale (Borg 1970). Training impulse (TRIMP) was calculated and employed as described in detail elsewhere (Zinner et al. 2018; Foster et al. 2001) by weighting the time spent in each zone (time in Zone 1 × 1, Zone 2 × 2, Zone 3 × 3).

*Baseline training:* During the 2-week baseline prior to the intervention, all participants performed their routine training, for which each individual TRIMP was calculated and defined as 100%.

### The 3-week mesocycles of training

On the basis of previous experience (Zinner et al. 2018), as well as to avoid non-functional overreaching and negative effects on health (Schwellnus et al. 2016; Soligard et al. 2016), we aimed to increase the training load for each individual by 10% per week. This increase was achieved either by elevating exercise intensity (HIIT), prolonging the training sessions (HVLIT) or performing more sessions each week (HFT).

*High-intensity interval training (HIIT):* Each HIIT-session consisted of a 10-min warm-up followed by 4 4-min sessions of high-intensity running (blood lactate > 4 mmol·l^−1^), with 3-min walking recovery between bouts. The participants performed either 2 or 3 such sessions each week, depending on their baseline characteristics.

*High-volume low-intensity training (HVLIT):* In this group, the duration of training sessions was set at 110% (week 1), 120% (week 2) and 130% (week 3) of the TRIMP calculated from the duration of each session during the baseline period. All participants performed two sessions per week, targeting a blood lactate concentration < 2 mmol·l^−1^.

*High frequency training (HFT): *In this group, the frequency of training sessions corresponded to 110% (week 1), 120% (week 2) and 130% (week 3) of the baseline TRIMP. Each session lasted as long as during the baseline period, with a running velocity that kept the concentration of blood lactate < 2 mmol·l^−1^.

### Statistical analysis

The responses of the three different groups, as well as intra-individual responses were subjected to statistical analysis as earlier (Zinner et al. 2018; Sylta et al. 2016).

The values for each variable for the different groups were compared employing repeated measures model ANOVA (with post-hoc analysis) carried out with the Statistica software package for Windows ® (version 7.1; StatSoft Inc., Tulsa, OK). The values obtained before and after each mesocycle for each individual group were compared as well. An alpha level of ≤ 0.05 was considered statistically significant.

To compare the responses of each individual to HIIT, HVLIT and HFT, a physiological adaptation index consisting of ${\dot{\text V}}{\text O}_{2 {\rm peak}}$, ${\dot{\text V}}{\text O}_{2 {\rm submax}}$ and V_2_ was calculated according to Zinner and co-workers (2018) who used a similar index. For this, we used ${\dot{\text V}}{\text O}_{2 {\rm peak}}$, ${\dot{\text V}}{\text O}_{2 {\rm submax}}$ and V_2_ since these variables are considered important physiological determinants explaining running performance (Midgley et al. 2007).

Since most of the parameters examined here are related to gas exchange, which involves a maximal error of 2% (Macfarlane and Wong 2012), all responses were categorized as none, moderate or high if altered by < 2%, 2–4% or > 4%, respectively.

## Results

### 24-h energy expenditure

Overall, each participant was monitored on average for 141.073 ± 8214 min (82.8 ± 4.5% of the total time) during the intervention period. The absolute and relative times spent in the different zones of energy expenditure during each period are documented in Table 1.

**Table 1:** Mean time spent by all participants in the various zones of energy expenditure during the mesocycles of training and “wash-out” periods

Mesocycle	Energy expenditure							
	< 1.5 MET		1.5 to 3 MET		3 to 6 MET		> 6 MET	
	*T* [min]	%	*T* [min]	%	*T* [min]	%	*T* [min]	%
HIIT: “wash-out”	9795 ± 3157	62.8	3721 ± 1358	24.5	1356 ± 892	8.2	611 ± 303	4.5
HIIT	18263 ± 3975	61.7	7591 ± 2152	25.8	2589 ± 1127	8.9	1044 ± 592	3.6
HVLIT: wash-out	10198 ± 2343	62.3	4032 ± 1034	24.9	1494 ± 638	9.3	552 ± 236	3.5
HVLIT	20604 ± 3433	65.0	7301 ± 2094	22.8	2665 ± 1530	8.3	1224 ± 794	3.9
HFT: wash-out	10556 ± 1800	64.3	4120 ± 805	25.2	1290 ± 638	7.9	444 ± 308	2.6
HFT	10117 ± 1585	64.4	7748 ± 2483	24.3	2419 ± 1338	7.5	1226 ± 909	3.8

*HIIT* high-intensity interval training, *HVLIT* high-volume low-intensity training, *HFT* high-frequency training

The mean time spent in the different zones of energy expenditure did not differ during the periods of HIIT, HVLIT or HFT training (*p* > 0.05) or during the “wash-out” periods (*p* > 0.05).

### Mesocycles of training

The TRIMP calculated for HIIT, HVLIT and HFT training increased by 150 ± 72, 182 ± 75 and 211 ± 136%, respectively, during the intervention, with no difference between these types of exercise (*p* > 0.05).

The number of sessions per week, as well as the average amount of time spent in zones 1, 2 and 3 and total training time per session are presented in Table 2. Table 3 summarizes all pre–post comparisons.

**Table 2:** The number of sessions per week, as well as the average amount of time spent in zones 1, 2 and 3 and total training time per session during each mesocycle of training

	Type of exercise		
	HIIT	HVLIT	HFT
Number of sessions per mesocycle	6.3 ± 0.9*	5.8 ± 0.9	11.8 ± 3.3*
Average time in zone 1 (%)	41.7 ± 0.7	45.1 ± 1.0	39.6 ± 0.7
Average time in zone 2 (%)	32.2 ± 0.8 +	50.1 ± 0.9	58.6 ± 0.5 +
Average time in zone 3 (%)	26.0 ± 0.7*	4.6 ± 4.1†	1.7 ± 2.7*†
Average total training time per session [min]	36.2 ± 0.9*	62.6 ± 0.9*	26.4 ± 1.0*

*HIIT* high-intensity interval training, *HVLIT* high-volume low-intensity training, *HFT* high-frequency training

^*^p < 0.05 HFT vs HVLIT vs HIIT

+*p* < 0.05 HFT vs HIIT

^†^*p* < 0.05 HVLIT vs HFT

**Table 3:** Effects of the different training interventions on performance and physiological parameters

Parameter	HIIT			HVLIT			HFT		
	Pre	Post	Δ%	Pre	Post	Δ%	Pre	Post	Δ%
Body mass [kg]	70.8 ± 11.9	70.8 ± 11.7	0.0	71.4 ± 11.6	70.9 ± 11.3	− 0.6	71.4 ± 11.7	71.2 ± 12	− 0.3
Peak oxygen uptake [ml·min^−1^·kg^−1^]	45.0 ± 5.1	45.4 ± 5.1	0.9 ± 4.0	44.8 ± 5.4	46 ± 5.5*	2.8 ± 4.0	44.3 ± 4.3	46.3 ± 4.3*	4.5 ± 5.5
Time-to-exhaustion [s]	1895 ± 223	1929 ± 222	1.8 ± 3.6	1918 ± 247	1922 ± 246	0.2 ± 3.1	1877 ± 205	1901 ± 230	1.3 ± 6.4
Submaximal oxygen uptake [ml·min^−1^·kg^−1^]	28.1 ± 2.1	27.6 ± 2.7	− 1.9 ± 5.0	28.1 ± 2.1	27.2 ± 1.5	− 2.1 ± 6.3	27.6 ± 0.9	27.8 ± 1.6	0.8 ± 5.5
Submaximal heart rate [b·min^−1^]	139 ± 15	138 ± 15	− 1.1 ± 3.1	139 ± 13	137 ± 12	− 1.0 ± 3.2	138 ± 13	134 ± 13*	− 3.0 ± 3.6
Velocity [m·s^−1^] with a blood lactate									
Concentration of 2 mmol·l^−1^	3.1 ± 0.4	3.1 ± 0.4	0.8 ± 1.2	3.1 ± 0.4	3.2 ± 0.5	1.0 ± 2.0	3.2 ± 0.3	3.1 ± 0.4	− 0.6 ± 1.2
Concentration of 4 mmol·l^−1^	3.6 ± 1.1	3.6 ± 0.3	0.2 ± 1.6	3.6 ± 0.3	3.6 ± 0.4	0.0 ± 1.9	3.6 ± 0.3	3.6 ± 0.4	0.1 ± 1.4

*HIIT* high-intensity interval training, *HVLIT* high-volume low-intensity training, *HFT* high-frequency training

^*^p < 0.05

Fat-free mass, time-to-exhaustion, ${\dot{\text V}}{\text O}_{2 {\rm submax}}$, V_2_ and V_4_ were not altered by any of the interventions (*p* > 0.05). ${\dot{\text V}}{\text O}_{2 {\rm peak}}$ was higher following HVLIT (+ 2.8%; *p* = 0.045) and HFT (+ 4.5%; *p* = 0.020), but not HIIT (+ 0.9%; *p* > 0.05), while HR_submax_ was reduced after HFT (− 3.0%; *p* = 0.03), but not after HIIT and HVLIT (*p* > 0.05). There was no difference between groups for any variable (*p* > 0.05).

**Fig. 2:**
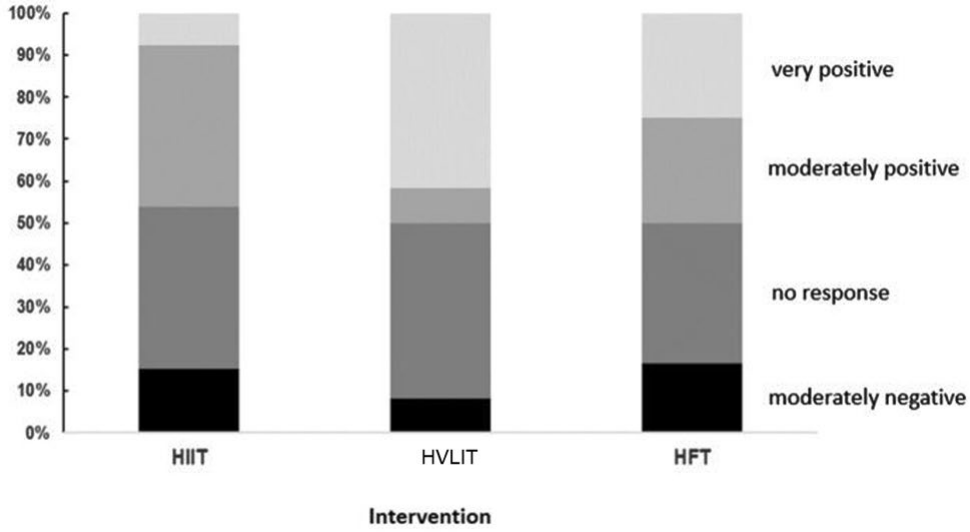
Relative mean responses (as indicated by the adaptation index) following the different interventions

As shown in Fig. 2, the relative amounts of very negative, moderately negative, no response, moderately positive and very positive responses to HIIT, HVLIT and HFT were 0%, 15.4%, 38.5%, 38.5%, 7.7%, and 0%, 7.6%, 46.2%, 7.7%, 38.5%, and 0%, 15.4%, 38.4%, 23.1%, 23.1%, respectively.

**Fig. 3:**
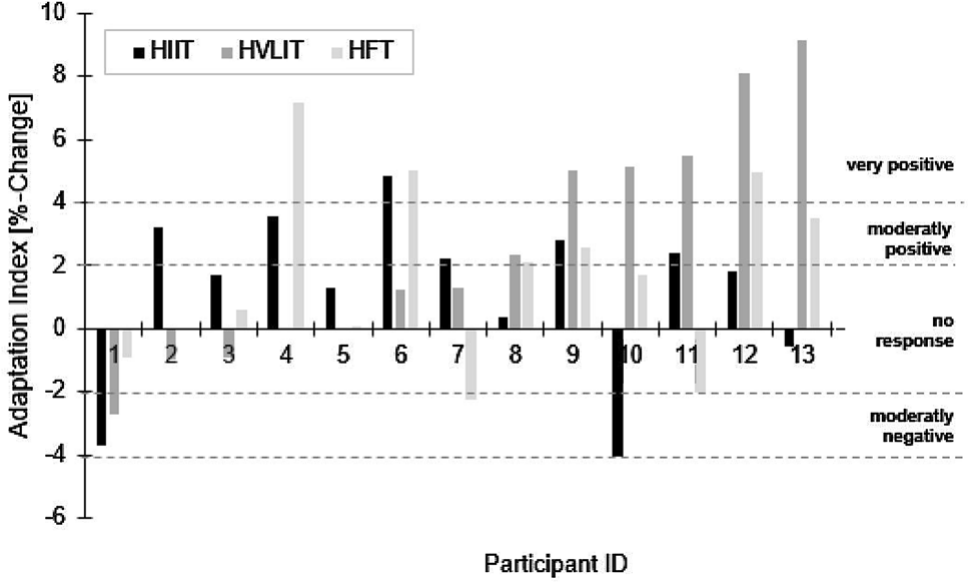
Adaptation index of each individual participant to HIIT, HVLIT or HFT

Figure 3 shows the adaptation index of each individual participant to HIIT, HVLIT or HFT.

Three participants (23%) demonstrated no positive response to any of the interventions; three (23%) responded positively to one; six (46%) exhibited a positive response to two interventions; and one (7%) responded positively to all three.

## Discussion

The aim of the present investigation was to assess the intra-individual physiological responses (i.e., peak oxygen uptake, velocity at the lactate threshold, running economy) and performance (TTE) of recreational runners to periods of HIIT, HVLIT and HFT. We hypothesized that different forms of mesocycles with a comparable load (i) induce different performance and physiological adaptations on group level and (ii) that each individual shows distinct adaptation to different mesocycles.

The main findings were as follows:

1. On group level ${\dot{\text V}}{\text O}_{2 {\rm submax}}$, V_2_, V_4_ and TTE did not change with HIIT, HVLIT or HFT (*p* > 0.05);
2. On group level, ${\dot{\text V}}{\text O}_{2 {\rm peak}}$ improved to the same extent following HVLIT (*p* = 0.045) and HFT (*p* = 0.02);
3. The number of moderately negative responders was higher following HIIT (15.4%); and HFT (15.4%) than HVLIT (7.6%);
4. The number of very high responders was higher following HVLIT (38.5%) than HFT (23%) or HIIT (7.7%);
5. 46% of the runners responded positively to two, while 23% did not respond to any type of mesocycle;
6. The magnitude of responses to HIIT, HVLIT and HFT was highly individual and no pattern was apparent.

Based on our own previous experience (Zinner et al. 2018) and to avoid non-functional overreaching and risk of injuries (Schwellnus et al. 2016; Soligard et al. 2016), the interventions were designed to involve an increase in training load of 10% TRIMP wk^−1^. However, the actual increase in TRIMP during the HIIT, HVLIT and HFT interventions was somewhat higher, i.e., 150 ± 72%, 182 ± 75% and 211 ± 136%, respectively. We speculate that our participants may have become overambitious while engaging in this type of experiment with professional support. The differences in TRIMP between mesocycles should be considered when interpreting our results as we cannot differentiate whether the variation originates from the different mesocycles or the different TRIMPS.

These substantial increases in TRIMP are somewhat higher than that reported by Zinner and colleagues (2018), who also investigated groups of recreational runners with a similar ${\dot{\text V}}{\text O}_{2 {\rm peak}}$. Their protocol involved one 4-week mesocycle of identical training by all three groups followed by a 3-week mesocycle of HIIT, HVLIT or both in combination (Zinner et al. 2018). These authors concluded that all three types of intervention resulted in similar improvement in the performance of recreational runners (Zinner et al. 2018). In contrast, our present findings indicate no difference in the change in TTE following 3 weeks of HIIT, HVLIT or HFT. 

Since habitual daily physical activity may influence the responses of recreational athletes to prescribed training (Hautala et al. 2012), we monitored the energy expenditure of each individual participant continuously throughout the intervention period and found no differences between the groups. However, we do not know when (i.e., during training or other daily activities) the energy was expended.

Here, ${\dot{\text V}}{\text O}_{2 {\rm peak}}$ was improved by HVLIT (+ 2.8%) and HFT (+ 4.5%), with no significant difference between these interventions in this respect. The fact that ${\dot{\text V}}{\text O}_{2 {\rm peak}}$ is improved by increasing the volume of exercise during each session and/or the frequency of sessions is a well-documented result of numerous central and peripheral adaptations, including alterations in stroke (Green et al. 1990) and plasma volume (Green et al. 1987), as well as in muscle blood flow (Coyle 1999).

We also observed that our participants increased their load significantly during the mesocycles of HVLIT and HFT, but not when performing HIIT. Since improvements in ${\dot{\text V}}{\text O}_{2 {\rm peak}}$ are dependent on the intensity of the exercise, the less pronounced elevation in TRIMP in response to HIIT may explain the absence of any change in ${\dot{\text V}}{\text O}_{2 {\rm peak}}$ in this case.

Moreover, we observed no changes in ${\dot{\text V}}{\text O}_{2 {\rm submax}}$ (a surrogate for running economy), V_2_ or V_4_, in line with the recent findings of Zinner and colleagues on recreational runners who performed 3 weeks of HVLIT or HIIT (Zinner et al. 2018). Running economy is dependent on a variety of factors, including biomechanical (e.g., relative stiffness of the musculotendinous system, stride length and frequency), neuromuscular (e.g., muscular strength) and morphological (e.g., fiber type distribution) parameters (Denadai et al. 2017). Since our protocol did not involve neuromuscular training and it may take months to change the musculotendinous system (Albracht and Arampatzis 2013), these findings are not surprising.

In the present case, HR_submax_ was decreased by − 3.0% (on average, approximately 4 beats·min^−1^) following HFT. Various forms of training reduce HR, both at rest and during submaximal exercise (Ekblom et al. 1968; Clausen et al. 1970; Andrew et al. 1966), with an average intra-individual variability of 4.1% (Achten and Jeukendrup 2003). In addition, submaximal heart rate is reduced by intensified training (Billat et al. 1999; Hedelin et al. 2000), which is sometimes attributed to overreaching (Achten and Jeukendrup 2003). Our routine interviews with all of our runners revealed no indications of feeling “washed-out”, tiredness, lack of energy, muscle and/or joint pain, or impaired immunity.

The changes in the mean adaption index that occurred here indicate that the numbers of positive and negative responders, as well as non-responders to HIIT, HVLIT and HFT were similar (Fig. 2), although more responded very positively to HVLIT (38.5%) than to HFT (23%) or HIIT (7.7%).

In this context, Zinner and co-workers (2018) observed more non-responders to HIIT than to HVLIT or HFT. In contrast to their study, we examined not only non-responders, but also negative responses, which were more pronounced in the case of HIIT (15.4%) and HFT (15.4%) than HVLIT (7.6%). On the individual level (Fig. 3), 46% of all our runners responded positively to two of the interventions. The magnitude of response to HIIT, HVLIT and HFT was highly individual and with our group of only 13 runners, no patterns were apparent.

Inter-individual differences in responses are important to emphasize, since 23% of all our participants did not respond to any type of the interventions, in line with other reports that as many as 20% do not respond to training (Timmons et al. 2010). We can only speculate as to why. In our experience three weeks of HIIT, HVLIT or HFT are sufficient to induce initial physiological adaptations, but maybe certain individuals require a greater amount (e.g., longer period) of exercise to achieve these adaptations. Indeed, Montero and Lundby (2017) showed that some of the 69% non-responders to less intense training (e.g., 60 min per week at a mean intensity of 65% of the individual’s maximal load), become responders when the training load was elevated to 240 and 300 min each week at the same mean intensity (Montero and Lundby 2017).

### Limitations

From a methodological point of view the number of participants in the present study was relatively small but in the range of similar studies employing a randomized cross-over design (Cesareo et al. 2019; Gillen et al. 2019; Fryer et al. 2019). Therefore, more subjects would have given more statistical power for the data interpretation, however the small sample size allowed us to monitor and control each training session and off-training activity. The results of the present responder analysis are only valid for the participants tested within this study and extrapolation to other individuals should be performed with caution. Since the present findings show no apparent pattern in individual response to different mesocycles we recommend applying different mesocycles to identify optimal individual training responses. Future research should evaluate individual response in a larger sample size.

Our participants were randomly assigned to the different mesocycles; therefore, we cannot identify preferable mesocycle sequences. Although we implemented wash-out periods after each mesocycle it might be possible that a preceding training mesocycle affected the outcomes of the following mesocycle.

As with almost every study applying a pre–post design and assessing performance and/or physiological parameters we cannot rule out if results are biased by within subject random variability (Atkinson et al. 2019). Yet, we reduced the within subject random variability in the present study by familiarizing participants with the testing protocols prior to any diagnostic testing and by keeping procedures and conditions constant during all testing. Future research should rule out bias by within subject random variability by allowing participants to undergo each mesocycle (i.e. HVLIT, HIT, HFT) twice.

Between each mesocycles we included 2 weeks without exercise to “wash-out” training effects before a new mesocycles. Although the 2-week wash-out period seems short we chose this 2-week period since a longer overall intervention, i.e. 3 × 3-weeks mesocycle including (longer) wash-out periods (i.e. > 2 weeks), too time-consuming most participants to adhere to the entire experiment. Future research should include longer wash-out periods and mesocycles.

## Conclusions

The findings of our cross-over study involving three 3-week mesocycles of HIIT, HVLIT or HFT performed by recreational runners indicate that on a group level, none of these interventions alters ${\dot{\text V}}{\text O}_{2 {\rm submax}}$, V_2_,V_4_ or TTE, while HVLIT and HFT improve ${\dot{\text V}}{\text O}_{2 {\rm peak}}$.

The changes in the mean adaption index revealed similar numbers of overall positive and negative responders, as well as of non-responders to HIIT, HVLIT and HFT, with, however, more who responded very positively to HVLIT (38.5%) than to HFT (23%) or HIIT (7.7%).

Moreover, 46% of our runners responded positively to two of the interventions, while 23% did not respond to any. The magnitude of responses to HIIT, HVLIT and HFT was highly individual and no patterns were apparent.

## Data Availability

Data are available from the corresponding author on reasonable request.

## Abbreviations

ANOVA: Analysis of variance
HR: Heart rate
HIIT: High-intensity interval training
HFT: High frequency training
HVLIT: High-volume low-intensity training
MET: Metabolic equivalent of task
$${\dot{\text V}}{\text O}_{2}$$: Oxygen uptake
V_2_: Running velocity at blood lactate concentrations of 2 mmol·l^−^^1^
V_4_: Running velocity at blood lactate concentrations of 4 mmol·l^−^^1^
TTE: Time-to-exhaustion
TRIMP: Training impulse
